# Continuing nursing education actions in the face of homophobia: an integrative review

**DOI:** 10.1590/0034-7167-2023-0094

**Published:** 2024-03-11

**Authors:** Milka Gabrielle de Lira Nóbrega West, Ednaldo Cavalcante de Araújo, Cláudia Margareth de Lira Nóbrega Vilar, Maria Amanda Lima Batista, Danilo Martins Roque Pereira, Adrian Thaís Cardoso Santos Gomes da Silva

**Affiliations:** I Universidade Federal de Pernambuco. Recife, Pernambuco, Brazil; II Governo do Estado de Pernambuco, Secretaria de Saúde. Recife, Pernambuco, Brazil

**Keywords:** Sexual and Gender Minorities, Nursing, Homophobia, Continuing Education, Training of Human Resources in Health, Minorías sexuales y de género, Enfermería, homofobia, Educación contínua, Formación de Recursos Humanos en Salud, Minorias Sexuais e de Gênero, Enfermagem, Homofobia, Educação Continuada, Capacitação de Recursos Humanos em Saúde

## Abstract

**Objectives:**

to analyze continuing nursing education actions in the scientific literature in the face of homophobia.

**Methods:**

an integrative literature review with structured search in June 2022 in eight databases, using the descriptors Nursing Education, Homophobia, Sexual and Gender Minorities. Final sample consisted of six primary studies.

**Results:**

continuing nursing education actions are supported by strategies such as use of teaching materials, lectures, case studies and focus groups, addressing content such as gender identity issues and affective-sexual orientation, health disparities and their relationship with homophobia in healthcare settings.

**Final considerations:**

carried out in various healthcare settings, continuing education actions proved to be successful in raising nurses’ awareness in facing homophobia in health services, however, their expansion is necessary to create health spaces that meet the specific needs of these people.

## RESUMO

**Objetivos:**

analisar na literatura científica ações de educação permanente da enfermagem frente à homofobia.

**Métodos:**

revisão integrativa da literatura com busca estruturada em junho de 2022 em oito bases de dados, utilizando os descritores *Nursing Education, Homophobia, Sexual and Gender Minorities*. Amostra final foi composta por seis estudos primários.

**Resultados:**

as ações de educação permanente da enfermagem apoiam-se em estratégias como utilização de materiais didáticos, palestras, estudos de casos e grupos focais, com abordagem de conteúdos como questões identitárias de gênero e de orientação afetivossexual, disparidades de saúde e sua relação com a homofobia nos locais de cuidados de saúde.

**Considerações finais:**

realizadas em variados cenários de cuidados de saúde, as ações de educação permanente mostraram-se exitosas quanto à sensibilização dos enfermeiros no enfrentamento da homofobia nos serviços de saúde, sendo necessária, no entanto, sua ampliação para a criação de espaços de saúde que atendam às necessidades específicas dessas pessoas.

## RESUMEN

**Objetivos:**

analizar las acciones de educación continua en enfermería en la literatura científica frente a la homofobia.

**Métodos:**

revisión integrativa de la literatura con búsqueda estructurada en junio de 2022 en ocho bases de datos, utilizando los descriptores Nursing Education, Homophobia, Sexual and Gender Minorities. La muestra final estuvo compuesta por seis estudios primarios.

**Resultados:**

las acciones de educación continua en enfermería se apoyan en estrategias como el uso de materiales didácticos, conferencias, estudios de casos y grupos focales, abordando contenidos como cuestiones de identidad de género y orientación afectivo-sexual, disparidades en salud y su relación con la homofobia en el ámbito de la salud.

**Consideraciones finales:**

realizadas en diversos ámbitos de atención de salud, las acciones de educación continua demostraron ser exitosas en la sensibilización de los enfermeros para enfrentar la homofobia en los servicios de salud, sin embargo, su ampliación es necesaria para crear espacios de salud que atiendan las necesidades específicas de estas personas.

## INTRODUCTION

The constructions of gender and sexuality in a way that differs from social norms constitute different patterns of sexual attitudes and behaviors from those recommended and practiced in different cultures. Because they do not follow the customarily ingrained assumption that all people are or should be heterosexual, whose identity can only correspond to the gender assigned at birth, ac-cording to the genitalia, such situations cause important social vulnerability. Cis heteronormativity is imposed through symbolic and physical violence, especially against those with gender differences, and is present in all social processes, including health services^(1)^.

Lesbian, gay, bisexual, transvestite, transsexual, transgender, queer, intersex, asexual/agender/aromantic, polysexual, non-binary and other people whose affective-sexual orientation and/or gender identity differs from social norms (LGBTQIAPN+) make up the group of sexual and gender minorities, representative in various sectors of society, and face limitations in receiving information, guidance and preventive healthcare or curative and palliative treatments, which compromise their health, not guaranteeing them human dignity due to stigma, discrimination and violence^(2, 3)^.

Widespread experiences of discriminatory care promote the distancing of LGBTQIAPN+ people and families from health services, a fact responsible for accentuating disparities. Healthcare spaces must be welcoming when providing care, which must be equitable and provide comprehensive care to LGBTQIAPN+ people, with professionals whose obligation is also to recognize the demands and specificities of the people in question. They must be free from prejudice, especially related to homophobia (LGBTQIAPN+phobia) -understood as actions of stigma, discrimination, omission and social exclusion, prejudice and/or violence motivated by affective-sexual orientation and/or gender identity other than cis heterosexuality^(3, 4)^.

As a consequence of homophobia, LGBTQIAPN+ people are more vulnerable to mental health disorders and disorders, such as anxiety, depression and suicidal ideation, in addition to the exacerbated consumption of tobacco, illicit psychoactive substances and alcoholic beverages, or even suffering from cardiovascular disorders and eating disorders, also due to receiv-ing inadequate healthcare. They are even more likely to exhibit behaviors that leave them vulnerable to contamination by the human immunodeficiency virus (HIV), viral hepatitis and other sexually transmitted infections (STIs)^(2, 3, 4, 5)^.

The inadequacy of care provided by healthcare providers, es-pecially nursing professionals, led by nurses, is due to the presence of explicit homophobia, demonstrated through hostility and even refusal of care and implicit, represented by discomfort in caring for LGBTQIAPN+ people. Therefore, the lack of understanding of social and health needs, related to professional training and scientific knowledge produced from a heterosexist perspective, must also be considered, which leads to low-efficiency healthcare and assistance^(4)^.

In the United States of America, the time dedicated to teaching content about the specificities and demands of people who belong to the sexual and gender minority group is estimated at two hours during the entire training in university nursing courses. In Brazil, despite the Brazilian National Policy for Comprehensive Health for Lesbian, Gay, Bisexual, Transvestite and Transgender People, whose guidelines are aimed at recognizing the social and health demands of this population and developing guidelines to implement actions whose objective is to reduce determinants of health inequalities and inequities through educational practices that promote knowledge, attitudes and provision of affirmative care, in the search to establish equity, the content approach on social and health issues of people who have different affective-sexual and/or gender identity orientations, in most nursing schools and faculties, is insufficient or non-existent, generating little or no knowledge about LGBTQIAPN+ people^(4, 5, 6, 7)^.

Thus, it is emphasized that such insufficiency in nursing training is reflected in their lack of knowledge and disrespect for professional ethical rights and duties, as they do not recognize that gender identity and affective-sexual orientation are decisive for health. Consequently, the establishment of a nurse-patient bond is disrupted or prevented. Without adequate training, nurses are unable to effectively screen patients for their health vulnerabilities, a contributing factor to the social marginalization of these people^(7)^.

Nursing teams in Brazil represent a significant percentage of personnel in health services, occupying a relevant position as part of the team and playing an important role in caring for people. In this regard, and with the purpose of providing competent and inclusive care, educational actions are necessary through continuing education programs with a view to providing the acquisition of knowledge meaningfully to achieve nurses’ professional capacity and personal development^(8)^.

It is observed that, given the contrast between health needs and the reality of care aimed at LGBTQIAPN+ people, health services must seek nurses’ professional development to improve the care provided, through the promotion of equity and the reduction of health disparities they face. Thus, educational actions contextual-ized in healthcare services regarding the demands and specificities of people with different gender identifications and affective-sexual orientation, with the purpose of providing access to inclusive, evidence-based and patient-centered information, can contribute to increasing understanding of health issues experienced by patients, promoting affirmative attitudes and skills, and allowing the breaking down of barriers, such as stigmas and homophobia in health services.

## OBJECTIVE

To analyze continuing nursing education actions in the sci-entific literature in the face of homophobia.

## METHODS

The study was conducted in accordance with national and international ethical guidelines. Ethical review and approval were waived due to use of data from secondary sources; therefore, the use of an Informed Consent Form was not applicable to carry out the present study.

This is an integrative literature review, a method that enables the identification, analysis and synthesis, in an orderly and sys-tematized way, of a phenomenon of interest, with a contribution to evidence-based practices, in addition to identifying possible gaps that can be resolved with the carrying out new research^(9)^.

The study was developed in six stages, in accordance with the proposal of Mendes *et al*.^(9)^. In the first stage, the guiding question of this investigation was formulated: how are continuing nursing educa-tion actions carried out in the face of homophobia? For elaboration, the PICo^(10)^ strategy was used, an acronym for “P”, which represents the nursing population. “I” symbolizes the intervention referring to continuing education actions to address different gender identities and affective-sexual orientation and to combat homophobia. “Co” reveals the context, which would be healthcare services.

During the second stage, evidence searches took place in June 2022, and data were collected through the MEDLINE, Web of Science (WoS), APA PsycINFO, SciVerse Scopus, Cumulative Index of Nursing and Allied Health (CINAHL), Cuiden, Latin American and Caribbean Literature in Health Sciences (LILACS) and Nursing Database (BDEnf ) databases. The descriptors selected from terms indexed in the Health Sciences Descriptors (DeCs) and Medical Subject Headings (MeSH terms) vocabularies were used, with the Boolean operator AND between Sexual and Gender Minorities, Nursing Education, Homophobia, carried out in three crosses, as described on Chart 1.

**Chart 1:** Database search strategies, 2022

Database	Search strategy	Number of articles
MEDLINE	(Nursing Education) AND (Homophobia)	59
	(Sexual and Gender Minorities) AND (Nursing Education)	337
	(Sexual and Gender Minorities) AND (Nursing Education) AND ((Homophobia)	19
CINAHL	(Nursing Education) AND (Homophobia OR Homonegativity OR Sexual Prejudice OR Antigay prejudice)	32
	(Sexual and Gender Minorities OR Homosexuality OR LGBT) AND (Nursing Education)	7
	(Sexual and Gender Minorities OR (Homosexuality OR LGBT) AND (Nursing Education) AND (Ho-mophobia OR Homonegativity OR Sexual Prejudice OR Antigay Prejudice)	1
Scopus	Title-ABS-Key (Nursing AND Education) AND Homophobia	49
	Title-ABS-Key (Sexual AND Gender AND Minorities) AND (Nursing AND Education)	133
	Title-ABS-Key (Sexual AND Gender AND Minorities) AND (Nursing AND Education) AND (Homophobia)	7
*Web of Science*	((ALL=(Nursing Education)) AND ALL=(Homophobia)	79
	((ALL=(Sexual and Gender Minorities)) AND ALL=(Nursing Education))	159
	((ALL=(Sexual and Gender Minorities)) AND ALL=(Nursing Education)) AND ALL=(Homophobia)	15
PsycInfo	Nursing Education AND Homophobia	50
	Sexual and Gender Minorities AND Nursing Education	82
	Sexual and Gender Minorities AND Nursing Education AND Homophobia	6
BDEnf	Nursing Education AND Homophobia	47
	Sexual and Gender Minorities AND Nursing Education	133
	Sexual and Gender Minorities AND Nursing Education AND Hompphobia	10
LILACS	Nursing Education AND Homophobia	-
	Sexual and Gender Minorities AND Nursing Education	1
	Sexual and Gender Minorities AND Nursing Education AND Homophobia	-
Cuiden	(*“Educación em Enfermería”*) AND (*“Identidad de género”*)	1

For the study, complete primary articles available in full, without time frame and that addressed continuing nursing education actions aimed at care without homophobia, published in Portuguese, English or Spanish, were included. Literature/ integrative/scope/systematic review studies, opinion studies, editorials, letters to the reader, summaries, brief communications, theses and dissertations were excluded. Duplicate articles were considered only once and as available in the database with the largest number of studies.

Initially, the studies were selected by exporting studies from databases to EndNote reference management software, thus extracting duplicates. Study selection occurred by pairs, blindly, and in the event of an impasse regarding study selection, a third reviewer participated to establish consensus, to meet the inclusion and exclusion criteria and minimize bias.

**Figure 1:**
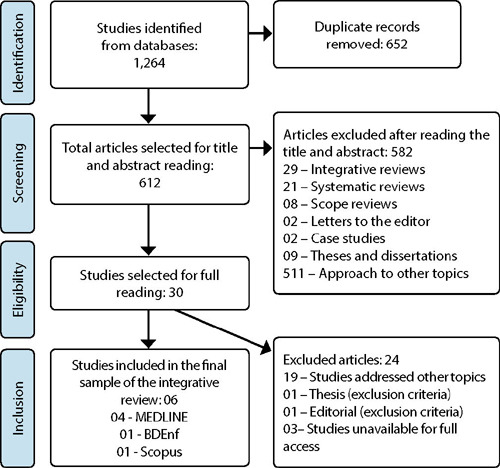
Study selection flowchart, 2022

After excluding duplicates, study titles and abstracts were read, considering those that corresponded to the topic. Selected studies were read in full, excluding those that in their textual body did not meet the inclusion criteria, obtaining the final sample of six articles. The route to reach the sample is described in the flowchart adapted from PRISMA^(11)^, as shown in Figure 1.

The third step consisted of defining the information to be ex-tracted from the selected studies/categorizing the studies. To this end, content analysis^(12)^ of data extracted from articles was carried out, which occurred from the use of an instrument proposed by JBI^(13)^ and the organization in Microsoft Word spreadsheet of title, year of publication, country of publication, objectives, methodology/ method, type of intervention, results and main findings related to research question. The studies were assesses regarding the level of evidence, according to the classification of the methodological approach^(10)^ as: randomized controlled clinical trial (Level II); clini-cal trial without randomization (Level III); cohort and case-control studies (Level IV); evidence derived from a single descriptive or qualitative study (Level VI). Systematic review or meta-analysis studies (Level I), systematic review studies of descriptive and qualitative studies (Level V) and opinion articles from authorities or reports from expert committees (Level VII) were not considered, according to the criteria of exclusion from this study.

In the fourth step, to carry out a critical assessment of meth-odological quality of selected studies, the JBI Critical Appraisal for Quasi-Experimental Studies instrument was used^(14)^, which assesses the methodological quality of quasi-experimental studies through the following questions: Q1 - Is it clear in the study what is the ‘cause’ and what is the ‘effect’ (i.e. there is no confusion about which variable comes first)? Q2 - Were the participants included in any comparisons similar? Q3 - Were the participants included in any comparisons receiving similar treatment/care, other than the exposure or intervention of interest? Q4 - Was there a control group? Q5 - Were there multiple measurements of the outcome both pre and post the intervention/exposure? Q6 - Was follow up complete and if not, were differences between groups in terms of their follow up adequately described and analyzed? Q7 - Were the outcomes of participants included in any comparisons measured in the same way? Q8 - Were outcomes measured in a reliable way? Q9 - Was appropriate statistical analysis used?

The JBI Critical Appraisal for Analytical Cross Sectional Stud-ies^(15)^ was also used, which evaluates the methodological quality of cross-sectional studies using the following criteria: Q1 - Were the criteria for inclusion in the sample clearly defined? Q2 - Were the study subjects and the setting described in detail? Q3 - Was the exposure measured in a valid and reliable way? Q4 - Were objective, standard criteria used for measurement of the con-dition? Q5 - Were confounding factors identified? Q6 - Were strategies to deal with confounding factors stated? Q7 - Were the outcomes measured in a valid and reliable way? Q8 - Was appropriate statistical analysis used?

According to the interpretation of the instruments, studies that reach scores of up to 49% are considered to have a high risk of bias and low methodological quality. Scores between 50 and 70% are considered to have a moderate risk of bias and method-ological quality. Studies with scores above 70% have a low risk of bias and high methodological quality. Thus, quasi-experimental studies^(16, 17, 18, 19, 20)^, considered in this integrative review, present high methodological quality and low risk of bias. The cross-sectional study^(21)^ has a risk of bias and moderate methodological quality, according to the description in Chart 2.

**Chart 2:** Assessment of methodological quality of selected studies through the JBI Critical Appraisal for Quasi-Experimental Studies and JBI Critical Appraisal for Analytical Cross Sectional Studies, 2022

JBI Critical Appraisal for Quasi-Experimental Studies checklist										
**Study**	**Q1**	**Q2**	**Q3**	**Q4**	**Q5**	**Q6**	**Q7**	**Q8**	**Q9**	**Score**
Hardacker CT, Rubinsteins B, Hotton A, Houlberg M^(16)^	Y	Y	Y	NA	Y	Y	Y	Y	Y	100.00%
Bristol S, Kostelec T, MacDonald R^(17)^	Y	Y	Y	NA	Y	Y	Y	Y	Y	100.00%
Schweiger-Whalen L, Noe S, Lynch S, Summer L, Adams E.^(18)^	Y	N	Y	NA	Y	Y	Y	Y	Y	88.88%
Wyckoff ED.^(19)^	Y	Y	Y	NA	Y	Y	Y	Y	Y	100.00%
Du Mont J, Saad M, Kosa SD, Kia H, MacDonald S^(20)^	Y	Y	Y	NA	Y	Y	Y	Y	Y	100.00%
**JBI Critical Appraisal for Analytical Cross Sectional Studies checklist**										
**Study**	**Q1**	**Q2**	**Q3**	**Q4**	**Q5**	**Q6**	**Q7**	**Q8**	-	**Score**
Kroning M, Green J, Kroning K^(21)^	Y	Y	Y	Y	N	Y	N	N	-	62.50%

*Y – Yes; N – No; NA - Not Applied*

The following steps, corresponding to interpretation of results and review/synthesis of knowledge preparation, respectively, will be described below.

## RESULTS

Selected studies were published in English and from 2014 to 2020, with a prevalence in 2018, with two studies. Five studies were carried out in the United States of America, and one study, in Canada, with a total sample of approximately 1,200 nurses who participated in continuing education actions on health issues for LGBTQIAPN+ people aimed at combating homophobia in health services. Five studies used quasi-experimental methodology, and one study was carried out using a cross-sectional design, according to the description in Chart 3.

**Chart 3:** Characteristics of selected studies, educational actions and contributions to face homophobia, 2022

Reference	Year/country	Design/number of participants	Interventions - educational actions	Outcomes - contributions to combating homophobia
Hardacker CT, Rubinsteins B, Hotton A, Houlberg M^(16)^	2014/United States of America	Multimethod study: methodological and quasi-experimental Number of participants: 848	- Development of curriculum on legal and health issues for LGBT older adults; - Training with lectures.	Increasing cultural competence during care for LGBT older adults.
Bristol S, Kostelec T, MacDonald R^(17)^	2018/United States of America	Quasi-experimental study Number of participants: 145	- E-learning with an approach to cultural competence; - Focus groups; - Use of videos about health and LGBT issues.	Improvements in care for LGBT patients.
Schweiger-Whalen L, Noe S, Lynch S, Summer L, Adams E.^(18)^	2018/United States of America	Quasi-experimental study Number of participants: 130	- Lectures on affirmative practices and LGBT health.	Increased knowledge and skills in creating a culture of inclusion.
Wyckoff ED.^(19)^	2019/United States of America	Quasi-experimental study Number of participants: 30	- Use of written material on health issues for LGBT people; - Lectures.	Improved cultural competence.
Du Mont J, Saad M, Kosa SD, Kia H, MacDonald S^(20)^	2020/Canada	Quasi-experimental study Number of participants: 47	- Case studies and focus groups on violence against trans people and the healthcare context.	Improved competence and skills to care for trans people who are victims of sexual assault.
Kroning M, Green J, Kroning K^(21)^	2017/United States of America	Cross-sectional study Number of participants: not reported	- Lectures on LGBT terminologies and healthcare; - Videos.	Implementation of best care practices for LGBT patients.

Data qualitative analysis made it possible to classify the analysis categories: Educational strategies used in continuing education; and Contents covered in continuing education actions. The diversity of areas of activity is evident, such as older adult care clinics, emergency clinics, reference departments for care for people who are victims of physical and/or sexual aggression, Intensive Care Units in which nurses who have received actions educational programs focused on health knowledge and issues of different gender identities and affective-sexual orientation, which can favor the provision of comprehensive and equitable care to LGBTQIAPN+ people, regardless of their stage of life or health demand.

Selected articles presented diverse approaches to carrying out continuing nursing education actions on health issues for LGBTQIAPN+ people and combating homophobia in health services, such as use of teaching materials, lectures, case stud-ies and focus groups, with successful experiences regarding the expansion of knowledge regarding the topic.

## DISCUSSION

### Educational strategies used in continuing education

Educational processes encompass the culture and historical-social context of which human work is a constituent. From the perspective that competent and welcoming care is a right for everyone and a duty for those who provide it, it is necessary to develop educational actions aimed at transforming behaviors and active participation of nurses as care providers. This perspective must be aimed at providing affirmative care and combating vio-lence experienced by LGBTQIAPN+ people. To modify behaviors, it is necessary to understand the context and the attribution of value given to homophobic actions carried out by those who should assist all people regarding health issues without distinctions^(22, 23)^.

In this way, education is conceived as a social practice built through participation, dialogue and meanings produced between subjects using methods and with the help of teaching materials^(24)^.

Use of teaching materials has been related to content conveying, and although of an informative nature, prior reading of materials relating to the topic to be discussed allows individual learning to be enhanced by optimizing time and making flexible times for taking knowledge regarding the topics covered. When associated with group educational strategies, it can strengthen well-founded discussions, making it possible to expand knowledge on issues of different gender identities and affective-sexual orientation^(25)^.

Use of printed, digital and audiovisual formats of materials allowed nurses to access information about gender and sexuality terminologies used in LGBT communities and the challenges of experiencing diversity in relation to binary gender norms, there-fore provided information that promoted better use of actions carried out in groups later^(17, 18, 19)^.

Another strategy used to disseminate knowledge, the lecture, is considered the most traditional and widespread expository method, used at all levels of education, which consists of an oral exposition of the chosen topic, previously structured and supported by teaching material. It has the advantage of passing on information and knowledge according to a logical structure, optimizing time and introducing new concepts - synthetically and globally - of the chosen topic. Furthermore, when dialogued, it allows spaces for active participation by those who also receive the information^(26)^.

The exposure of content on the specificities and demands of sexual and gender minority people through lectures is suggested as one of the proposals for including LGBT content to raise aware-ness and knowledge of health professionals, in all training stages, as an alternative to articulate and affirmative practices^(27)^. The most prevalent educational method was configured among the articles considered for the present study in terms of addressing issues related to LGBTQIAPN+ health in continuing education actions for nurses^(16, 19, 21)^.

Use of lectures as an educational strategy on health issues for LGBTQIAPN+ people in the studies analyzed promoted an increase in awareness and affirmative practices by nurses^(16, 19, 21)^. However, despite making it possible to be closer to the topic, just being present during the development of an educational activity, but without active participation, a fact often witnessed during lectures does not guarantee the desired learning process, as the construction of knowledge requires more than listening and memorizing what is transmitted^(28)^.

Thus, the adoption or combination of educational actions that involved the active participation of everyone, such as using case studies and focus groups, promoted greater impacts on learn-ing and the practice of welcoming, respectful, competent and communicative care by nurses in relation to LGBTQIAPN+ people, according to the results presented by studies analyzed^(17, 20)^. In the meantime, they were implemented in formal spaces for training human resources in health in countries, such as the United States of America, the United Kingdom and Kenya, methodological strategies whose objectives were to foster the discussion of topics related to LGBTQIAPN+ issues, in addition to promoting increased knowledge and practical skills for providing healthcare that considers the body in its entirety^(29)^.

The case study is understood as a didactic approach that aims to bring theory and practice closer together through the descrip-tion and understanding of the phenomenon and the context in which it is inserted^(30)^. Using such an approach to topics related to health issues of people of different gender identities and affective-sexual orientation enabled discussions about factors related to LGBTQIAPN+ people’s health, by providing an under-standing of the circumstances of homophobia and the effects on the provision of care by nurses, based on an understanding of the terminology used by trans people and the experiences of sexual abuse suffered by LGBTQIAPN+ people. Furthermore, it is important to highlight understanding and reflection regarding the stigma and discrimination to which trans people are exposed when seeking care in health services. Such changes in attitude, therefore, promote a significant increase in nurses’ skills and abilities to provide affirmative healthcare^(20)^.

In the role of an educational strategy that also encourages the active and contextualized participation of all those involved, using focus groups - aimed at promoting LGBTQIAPN+ people’s health and practices contrary to homophobia - becomes interesting, as its approach is based on reflection through participants’ speeches and is supported by the full participation of those involved in the educational process, as it allows the approach and involvement with health issues faced by LGBTQIAPN+ people and the active construction of ways of coping with homophobia.

Focus groups were used to understand higher education students’ social representations regarding sexual diversity and citizenship rights. They made it possible to understand the intrinsic relationship between the conception of gender and religious values in homophobic thinking construction in Brazil-ian society through knowledge of beliefs and contexts of people who practice homophobia, in the search for understanding the causes of violence against LGBTQIAPN+ people^(31)^, a relationship also identified by this educational strategy on health disparities and homophobia^(17, 20)^.

### Contents covered in continuing education actions

The scarce understanding of gender, in its broad meaning, goes beyond the binary construction of feminine or masculine identity based solely on biological factors, to the change in a constitution supported by multiple aspects, such as attitudes, expectations and behaviors, recognizing other gender identi-ties, such as trans and agender people and even cis women and men in the performance of roles different from those socially normalized. Sexuality, which goes beyond the meaning of hu-man reproduction and refers to affective, intimate and sexual attraction for another person, is generally related to the training curriculum and care practices of nurses marked by compulsory heterosexuality, causing a lack of information about identity aspects and issues of LGBTQIAPN+ people^(1, 32, 33)^.

Lack of knowledge about the terminology used to image and identify LGBTQIAPN+ people is perceived as one of the greatest barriers to providing competent care, and is related to the lack of welcoming of people by nurses. The LGBTQIAPN+ population has specificities and complexities, and encompasses many dif-ferent terminologies and manifestations, and to provide compe-tent care to people belonging to sexual and gender minorities, nurses must use the terminologies with which people recognize themselves. Terms should not be exclusive or comprehensive, and should consider the multiple overlapping possibilities of personal identification^(34)^.

By asking patients about their pronouns of choice and using them appropriately, people feel identified and understood^(34)^, a fact that can bring them closer to health services and reduce disparities^(35)^. Understanding and approaching the appropriate terminology to be used helped to promote effective communica-tion between nurses and health service users, avoiding prejudice against heterosexual language and focusing on using gender-affirming language^(17, 18, 19, 20, 21)^. In this understanding, the findings of this review addressed gender and affective-sexual orientation terminologies as essential topics for affirmative health practices free from homophobia^(16, 17, 18, 19, 20, 21)^.

However, in order for tackling homophobia in health services to have a greater impact and be expanded to other social spaces, it is necessary to provide, through continuing education actions, critical dialogues that promote not only the correct way of ad-dressing someone, but also questioning the process of stigma and discrimination responsible for declassifying people from sexual and gender minorities^(4)^.

Furthermore, the educational approach to knowledge and reflection on health disparities, commonly experienced by LG-BTQIAPN+ people, in this context, is essential so that there is real planning and changes in practices aimed at care and promote prejudice-free healthcare^(16, 17, 18, 19, 20, 21)^.

Mental health is one of the main areas of health disparity, especially gay people and trans, transvestite and bisexual men and women, who often use tobacco, abuse of illicit psychoactive substances and alcohol as strategies for coping with the stigma and prejudice experienced. Social satisfaction can often be related to the consumption of alcohol and illicit psychoactive substances. At moderate levels of social satisfaction, the consumption of these substances is considered high, with an average risk of dependence in 46.9% of LGBTQIAPN+ people who use this means to escape the lack of social support and stress due to prejudice. When consump-tion occurs immediately before or during sexual practices (chemical sex), people are also exposed to vulnerable behavior in terms of the risk of contamination by HIV, viral hepatitis and other STIs, due to the loss of decision-making capacity in the use of condoms and the perception of risk of contamination, heightened by the lack of guidance and access to information and combined prevention methods in health services^(36, 37)^.

The complexity of psychological distress, especially among LGBTQIAPN+ adolescents, also requires mental healthcare pro-viders to take actions and strategies that enable acceptance and coping with the violence experienced and, due to the multiplic-ity of demands, educational actions on effective treatment and services for these people become even more necessary^(38)^.

In addition to the stigma and violence suffered, the invisibility of the specific demands of people of different gender identities and affective-sexual orientation in health services causes these people to withdraw from institutions and actions and decisions to protect and care for their own health, generating even more health disparities for LGBTQIAPN+ people.

Inadequate perception, due to lack of actions and guidance by health professionals, regarding the risks of contamination by HIV, other STIs and viral hepatitis, in addition to care that does not correspond to lesbian women’s demands due to unpreparedness and even discomfort by health professionals, discourages using condoms, combined prevention measures and gynecological care. Thus, lesbian women are more prone to developing gyne-cological cancer, such as breast cancer and cervical cancer, due to less demand for health services, due to discriminatory practices after declaring sexual orientation other than heteronormativity: from the lack of confidentiality to exposure of socially devalued intimacy and judgments, including those of a religious nature^(39, 40)^.

Just like invisibility, the lack of qualifications of health profes-sionals in relation to the care of transgender people promotes greater exposure of these people to mechanisms and substance use, in the search for the desired body modification, such as uncon-trolled and inappropriate use of hormones and self-application of non-aesthetic silicone, increasing the morbidity and mortality of these people^(41)^. Nurses, when developing professional skills and competencies, must know and value the real needs of those who receive their care, through knowledge and reflections on people’s health issues regarding their gender identity and affective-sexual orientation, by establishing a welcoming environment that favors the creation of bonds in health services, spaces currently marked by stigma, fear and homophobia.

### Study limitations

During the data collection period, few studies were found in the established databases aimed at carrying out continu-ing nursing education actions involving the topic in question, mainly published in Brazil, which did not allow us to understand the reality of educational practices to combat homophobia in health services covering nurses’ work, especially those working in Brazilian health services.

The acronym LGBTQIAPN+, which encompasses people of different sexual orientations and gender identities in a broader sense, is currently represented, in the findings for this study, by the shorter acronym, LGBT. Even though there are differences in the expression to represent these people, their health issues addressed in studies refer to the same meanings and challenges for carrying out inclusive healthcare and practices.

### Contributions to nursing, health or public policy

The health inequities suffered by LGBTQIAPN+ people, caused by prejudice and lack of approach to issues related to health and specific demands, make educational actions strategies for knowledge, discussion and awareness among nurses, especially if developed in healthcare provision spaces, with the aim of en-abling professional development and, consequently, promoting effective care for these people and combating homophobia in healthcare services.

Thus, this review can promote the expansion of the debate on the topic, by involving nurses in the discussion with the aim of reducing health inequities experienced by LGBTQIAPN+ people.

## FINAL CONSIDERATIONS

Evidence in the scientific literature shows that continuing nursing education actions aimed at combating homophobia use, among the main educational strategies, printed, digital and audiovisual teaching materials, case studies, focus groups and, above all, lectures. The latter was the most prevalent educational method among the articles considered for this study regarding the approach to LGBTQIAPN+ issues and health, which allows dissemi-nating knowledge in a structured way, enabling the foundation on the topic by providing the opportunity to discuss the possibility of critical reflections regarding health inequities and homophobic behavior aimed at LGBTQIAPN+ people in health services.

The studies analyzed reveal the fundamental approach to content related to gender terminology and health disparities caused by homophobia in health services, which demonstrates the gap in the professional development of nurses regarding healthcare for LGBTQIAPN+ people during the initial training process. However, when explored during continuing education actions, such topics promote, in nurses’ experience, an increase in knowledge and affirmative practices and awareness regarding facing homophobia in healthcare settings. Therefore, it is still necessary, however, to expand these actions to create health spaces that meet the specific needs of these people.
